# Bone Mesenchymal Stem Cells Contribute to Ligament Regeneration and Graft–Bone Healing after Anterior Cruciate Ligament Reconstruction with Silk–Collagen Scaffold

**DOI:** 10.1155/2021/6697969

**Published:** 2021-04-23

**Authors:** Fanggang Bi, Yangdi Chen, Junqi Liu, Wenhao Hu, Ke Tian

**Affiliations:** ^1^Department of Orthopedic Surgery, The First Affiliated Hospital of Zhengzhou University, No. 1 Jianshe East Road, Zhengzhou, China 450001; ^2^Henan University of Chinese Medicine, No. 156 Jinshui East Road, Zhengzhou, China 450001; ^3^Department of Radiation Oncology, The First Affiliated Hospital of Zhengzhou University, No. 1 Jianshe East Road, Zhengzhou, China 450001; ^4^Spine Division, Department of Orthopedics, The Fourth Medical Center of PLA General Hospital, No. 51 Fucheng Road, Beijing, China

## Abstract

Anterior cruciate ligament (ACL) reconstruction was realized using a combination of bone mesenchymal stem cells (BMSCs) and silk–collagen scaffold, and an in vivo evaluation of this combination was performed. By combining type I collagen and degummed silk fibroin mesh, silk–collagen scaffolds were prepared to simulate ligament components. BMSCs isolated from bone marrow of rabbits were cultured for a homogenous population and seeded on the silk–collagen scaffold. In the scaffold and BMSC (S/C) group, scaffolds were seeded with BMSCs for 72 h and then rolled and used to replace the ACL in 20 rabbits. In the scaffold (S) group, scaffolds immersed only in culture medium for 72 h were used for ACL reconstruction. Specimens were collected at 4 and 16 weeks postoperatively to assess ligament regeneration and bone integration. HE and immunohistochemical staining (IHC) were performed to assess ligament regeneration in the knee cavity. To assess bone integration at the graft–bone interface, HE, Russell–Movat staining, micro-CT, and biomechanical tests were performed. After 4 weeks, vigorous cell proliferation was observed in the core part of the scaffold in the S/C group, and a quantity of fibroblast-like cells and extracellular matrix (ECM) was observed in the center part of the graft at 16 weeks after surgery. At 4 and 16 weeks postoperatively, the tenascin-C expression in the S/C group was considerably higher than that in the S group (4 w, *p* < 0.01; 16 w, *p* < 0.01). Furthermore, bone integration was better in the S/C group than in the S group, with histological observation of trabecular bone growth into the graft and more mineralized tissue formation detected by micro-CT (4 w, bone volume fraction (BV/TV), *p* = 0.0169, bone mineral density (BMD), *p* = 0.0001; 16 w, BV/TV, *p* = 0.1233, BMD, *p* = 0.0494). These results indicate that BMSCs promote ligament regeneration in the knee cavity and bone integration at the graft–bone interface. Silk–collagen scaffolds and BMSCs will likely be combined for clinical practice in the future.

## 1. Introduction

The anterior cruciate ligament (ACL) is a main structure that maintains stability of the knee [1]. As a common athletic injury, ACL rupture can cause serious damage such as knee joint instability, injury to other ligaments, dislocation, and osteoarthritis [2, 3]. ACL reconstruction is currently considered the gold standard for treating ACL rupture, and grafts including autografts, allografts, and synthetic grafts are used for this purpose [4–6]. However, these grafts have some limitations. Shortcomings of autografts include long surgery time, donor site complications, long rehabilitation time, and decrease in knee range of motion [7]. Disadvantages of allografts include higher cost, higher infection rates, and a higher failure rate compared to autografts [8]. The complications of permanent synthetic grafts include osteoarthritis, chronic synovitis, foreign-body response, and long-term rupture [9]. Bone mesenchymal stem cells (BMSCs) and ligament tissue engineering have become promising techniques for addressing these drawbacks.

To reconstruct the ACL well, tissue engineering needs to meet the following criteria: provide immediate joint stability after surgery, assure good ligament regeneration in the knee cavity as the engineered tissue gradually degrades and diminishes, and establish good bone integration at the graft–bone interface for long-term stability after surgery [10]. In a previous study, we designed a graft by combining collagen matrix with knitted degummed silk fibroin to reconstruct the ACL in a rabbit model [11]. The silk–collagen scaffold was discovered to have good biocompatibility and biomechanical properties [12]. However, in the early postoperative period, limited ingrowth of the newly regenerated connective tissue in the knee cavity restricted ligament regeneration, and bone tissue in the bone tunnel disrupted graft–bone healing [13, 14].

BMSCs are pluripotent cells and have become a very important source of cells for cell therapy and engineered tissue repair [15]. Their multiple differentiation potential for therapeutic application when implanted with biodegradable scaffolds has been demonstrated in several previous studies [16–19]. Although which cell types initiate and regulate the ligament regeneration and graft-bone healing process has not been clarified [20], it seems that BMSCs in the marrow from the bone tunnel promote ligament regeneration and repair at the graft–bone interface [21]. Lim et al. demonstrated that the failure load and stiffness of MSC-enhanced hamstring tendons were obviously greater at 8 weeks after ACL reconstruction surgery in a rabbit model [22]. According to Soon et al., MSCs may form an intermediate fibrocartilage zone between bone and the allograft tendon after reconstruction surgery [23].

No unified and widely accepted approach has been available to guide how BMSCs are applied. BMSC application approaches include local injection [24], BMSCs sheet technology [25], combination with fibrin glue or collagen gel [17, 26], and implantation on a scaffold for tissue engineering [27, 28]. Implanting BMSCs on a tissue-engineering scaffold seems more reliable because of its small distraction on the growing status and environment of BMSCs. Based on this background, we seeded BMSCs on a silk–collagen scaffold, attempting to determine whether BMSCs could promote ligament regeneration in the knee cavity and graft–bone healing. In the present study, a rabbit ACL reconstruction model with silk–collagen scaffold with or without BMSCs was established. We hypothesized that BMSCs could improve knee ligament regeneration and bone integration at the graft–bone interface, as demonstrated by histological assessment, micro-CT, and a biomechanical test.

## 2. Materials and Methods

### 2.1. Scaffold Preparation

The raw silk fibers were provided by Zhejiang Cathaya International, Ltd. The degumming process was completed using 0.02 M Na_2_CO_3_ (100°C for 60 min, 3 times) to extract sericin, as described in a previous study [11]. Isolation and purification of the collagen matrix from pigs' Achilles tendons were performed with dilute acid and neutral salt extractions [29]. The knitted silk mesh extracted from sericin was soaked in acidic collagen solution (type I, pH 3.2, w/v 1%), freeze-dried (–80°C for 12 h followed by Heto PowerDry LL1500 for 24 h), and subjected to dehydrothermal crosslinking in a vacuum oven (30 mTorr, 105°C for 24 h) [11]. Observation of the surface microstructures of the raw silk, degummed silk, and silk–collagen scaffold was performed using a scanning electron microscope (SEM). Finally, cobalt-60-sterilized silk–collagen scaffolds were prepared for the following evaluations.

### 2.2. Isolation, Culture, and Identification of BMSCs

Bone marrow aspirates extracted from New Zealand White rabbits (2.5 ± 0.2 kg, 12 weeks old) were used to isolate and culture BMSCs, as previously described [17]. Mononuclear cells were gathered in Ficoll–Hypaque gradient (Sigma) after centrifugation and then suspended in cell culture medium containing 10% fetal bovine serum (FBS, Gibco). As the culture medium changed, the suspended cells were removed after culture at 37°C in 5% CO_2_ for 72 h. When the adherent cells reached 70–80% confluence, subculture was performed. After culture for 2 weeks, a homogenous BMSC population was obtained, and the third passage was collected and seeded on the silk–collagen scaffold. BMSCs adherent on the scaffold were observed by SEM after being seeded for 72 h. The osteogenic, adipogenic, and chondrogenic differentiation abilities of passage 3 cells were identified after culture with special inducing media (Gibco) for 3 weeks. Finally, alizarin red (Sangon), oil red O (Sangon), and alcian blue staining (Sangon) were performed according to the manufacturer's protocols.

### 2.3. Flow Cytometry

To confirm the homogeneous property of passage 3 BMSCs cultured at 2 weeks, a characterization for stemness markers was performed. Sheep anti-rabbit antibodies of CD29, CD73, CD105, and phycoerythrin-(PE-) labeled IgG secondary antibody were purchased from the eBioscience (San Diego, CA). Approximately 3 × 10^5^ cells were harvested and resuspended in 100 *μ*L phosphate-buffered saline (PBS). Cells were incubated with primary antibodies of CD29 (1 : 100), CD73 (1 : 100), and CD105 (1 : 100) for 1 hour at 4°C. Subsequently, the cells were washed with PBS for 3 times, and the supernatant was discarded by centrifugation at 250 × g for 5 minutes. And the cells were resuspended in 100 *μ*L PBS and incubated with PE-labeled secondary sheep antibody (1 : 200) for 40 minutes at 4°C in the dark. The cells were washed with PBS for 3 times, and the supernatant was discarded by centrifugation at 250 × g for 5 minutes. The cells were immediately tested on the machine (BD LSRFortessa) after resuspended with 400 *μ*L PBS. The data were analyzed with FlowJo 10.0 software.

### 2.4. Animal Model Study Design

**Figure 1:**
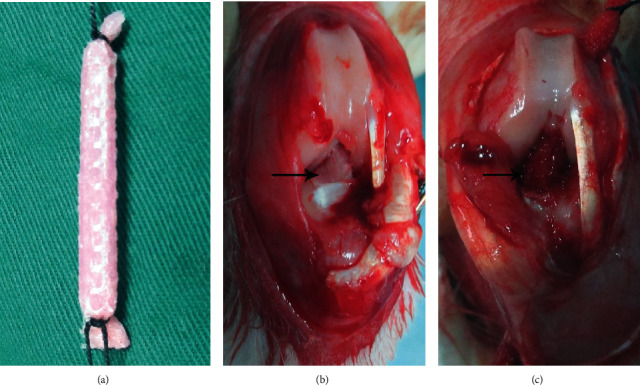
(a) After coculture with BMSCs in the S/C group and immersion only in culture medium in the S group for 72 h, the scaffold was rolled for use as a graft to replace the native ACL in a rabbit model. (b) General observation of the native ACL: the arrow points to the native ACL. (c) General observation of the knee after ACL reconstruction with the scaffold: the arrow points to the implanted graft.

The present study used 40 male New Zealand white rabbits provided by Hualan Biology (2.5–3.0 kg, 12 weeks old, certification No.: SYDW20190409). The ethics committee of the First Affiliated Hospital of Zhengzhou University approved the experimental protocol (ethics review No.: 2020-KY-012). Two equal-numbered groups (scaffold group, S; scaffold and BMSCs group, S/C) were formed by dividing the rabbits at random, and ACL reconstruction was carried out in the knee of the left hind leg. In the S group, silk scaffolds were immersed in culture medium for 72 h, whereas in the S/C group, silk scaffolds were seeded with BMSCs for 72 h; then both types of scaffold were rolled and used for ACL reconstruction (Figure 1(a)). At 4 and 16 weeks after the operation, 10 rabbits from each group were sacrificed. Five specimens in each group were assessed for ligament regeneration by hematoxylin and eosin (HE) staining and immunohistochemical (IHC) staining and for bone integration at the graft–bone interface by HE and Russell–Movat (RM) staining. Graft–bone healing was assessed in the remaining specimens (*n* = 5) using micro-CT and the biomechanical test.

### 2.5. Surgical Procedure

ACL reconstruction was carried out under strict aseptic conditions, and all operations were performed by one person (Bi). After general anesthesia was achieved by pentobarbital (Kyoritsu-seiyaku, 30 mg/kg body weight), the surgical area was shaved, disinfected, and covered. Exposure of the knee cavity was achieved by a 3 cm incision along the patellar tendon, and then the native ACL was removed (Figure 1(b)). A 2.0 mm Kirschner wire was used to make tunnels in the femur and tibia. The graft was inserted through the bone tunnels, and its ends were attached to the surrounding soft tissue and the periosteum with 1–0 Ethibond suture (Figure 1(c)). Then, the rabbits were raised in their cages without restriction after surgery.

### 2.6. Ligament Regeneration Assessment

After collection, the tibia–graft–femur complexes (*n* = 5 per group at each point in time) were immediately put in paraformaldehyde (4%; Sangon) for 24 h. The graft in the knee cavity was collected, dehydrated, and embedded. After sectioning, the slices were stained with HE. Ligament regeneration was analyzed by immunohistochemistry staining for tenascin-C. Image-Pro Plus 6.0 software (IPP6.0) was used to calculate the average immunoreactivity density of tenacin-C in the graft.

### 2.7. Graft–Bone Healing Assessment

After the graft was dissected from the tibia–graft–femur complex, bone integration at the graft–bone interface was assessed using the remaining bone samples. The bone samples were decalcified by ethylenediaminetetraacetic acid (EDTA; 10%) until they could be easily sectioned with a blade. The samples were sectioned after dehydration and embedding, and the slices were stained with HE and RM to evaluate bone integration.

### 2.8. Micro-CT Evaluations

The tibia–graft–femur complexes (*n* = 5 per group at each point in time) were prepared for micro-CT scan (36 *μ*m thickness; Skyscan 1176, Bruker, Antwerp, Belgium) and immediately stored at –80°C after collection. The specimens were placed in a refrigerator (4°C) overnight to thaw before testing. Detection of mineralized tissue regeneration at the graft–bone interface was performed using radiograph images. Calculation of the bone mineral density (BMD), trabecular number (Tb.N), bone volume fraction (BV/TV), trabecular thickness (Tb.Th), and trabecular separation (Tb.Sp) of a 2.0 mm diameter cylinder scope including the graft–bone interface was carried out by 3-dimensional standard microstructural analyses [30].

### 2.9. Biomechanical Test

The next step was to carry out the biomechanical test. The tibia–graft–femur complex (*n* = 5 per group at each point in time) was created by dissecting all soft tissue around the knee joint except for the graft. The femur and tibia were screwed into custom-made steel pipes, and the steel pipes were secured to an Instron 553A biomechanical testing system (Instron). The crosshead speed of the tensile load during the biomechanical test was 5 mm/min. The elongation (mm) and failure load (N) were documented, and the slope of the recorded curve indicated stiffness (N/mm). The tibia–graft–femur complexes were kept moist with normal saline.

### 2.10. Statistical Analyses

The data collected in the present study are expressed as mean ± standard deviation (SD). SPSS 16.0 software was used for the statistical analyses. Differences were considered statistically significant at *p* < 0.05. Independent-sample *t*-tests were used to detect differences between groups.

## 3. Results

### 3.1. SEM Observation

**Figure 2:**
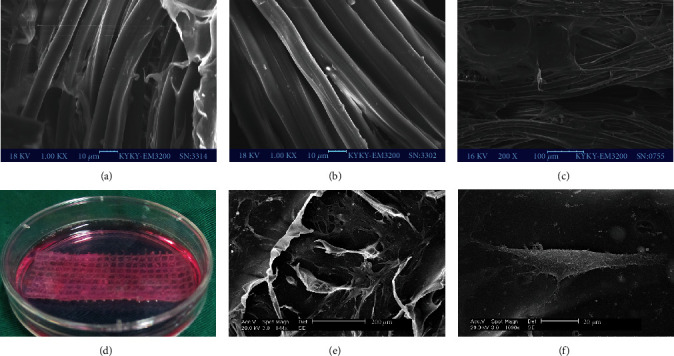
(a) The microstructure of silk fibers before degumming: the surface is coated with the glue-like protein sericin. (b) Under SEM, the surface of silk fibroins becomes smooth after complete degumming; the average diameter is about 10 *μ*m. (c) The microstructure of silk-collagen scaffold after the dehydrothermal crosslinking process: the collagen sponge permeated into the rings of knitted mesh. (d) BMSCs were seeded on the scaffold and cocultured for 72 h for further use. (e) BMSCs exhibited vigorous proliferation on the scaffold. (f) BMSCs retained good cellular morphology on the scaffold observed by SEM.

The surface of raw silk fibers was irregular due to the sericin coating on the silk fibroin (Figure 2(a)). The silk fibroins, which were about 10 *μ*m in diameter and had a smooth surface, were visible after complete degumming (Figure 2(b)). After the process of freeze-drying and dehydrothermal crosslinking, the collagen sponge distributed on the silk fibroin surface entered into the rings of knitted mesh, resulting in a fuzzier surface (Figure 2(c)). BMSCs adhered to the collagen surface after being seeded onto the scaffold for 72 h in Petri dishes (Figure 2(d)) and maintained good cellular morphology (Figures 2(e) and 2(f)).

### 3.2. Identification of BMSCs

**Figure 3:**
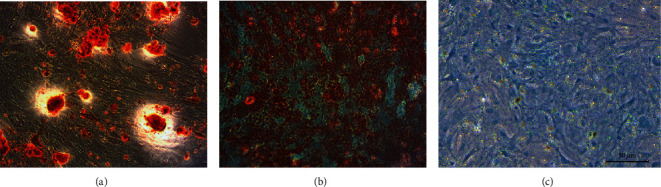
Representative images from alizarin red (a), oil red O (b), and alcian blue (c) staining to detect the osteogenic, adipogenic, and chondrogenic differentiation abilities of passage 3 cells.

**Figure 4:**
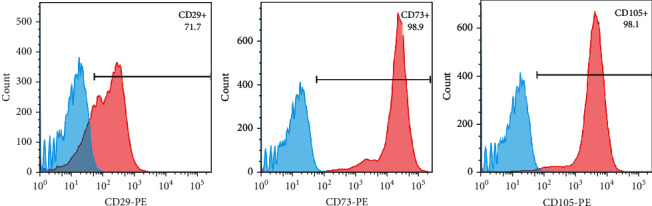
The third passage cells had high expression of CD29 (71.7%), CD73 (98.9%), and CD105 (98.1%).

Alizarin red, oil red O, and alcian blue staining were performed after cells were cultured in the inducing medium for 3 weeks. Mineralized nodules, lipid droplets, and green cytoplasm were observed under the microscope after staining with alizarin red, oil red O, and alcian blue (Figure 3). The results of flow cytometry showed that the third passage cells had high expression of CD29 (71.7%), CD73 (98.9%), and CD105 (98.1%) (Figure 4).

### 3.3. Ligament Regeneration Assessment

**Figure 5:**
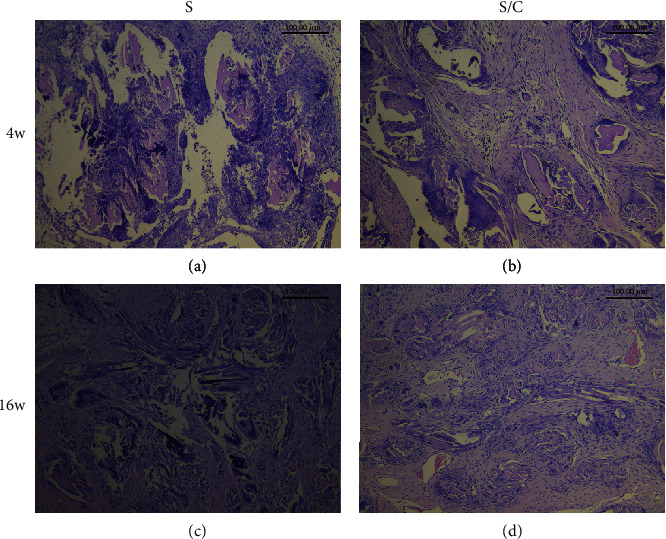
At 4 weeks after surgery, HE staining of grafts in the knee cavity in the S group (a) revealed few cells, whereas considerable cells were observed in the core part of the graft in the S/C group (b). At 16 weeks after surgery, fibroblast-like cells became more regular and denser in the S/C group (d) than in the S group (c).

**Figure 6:**
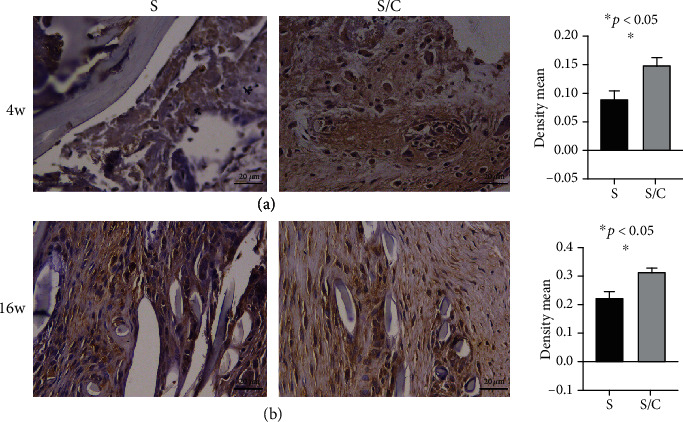
Immunohistochemistry staining of grafts in the knee cavity specific for tenascin-C in the S group and S/C group to assess ligament regeneration: the density mean of immunoreactivity was higher in the S/C group than in the S group at 4 weeks (a) and 16 weeks (b) after the operation.

Cellular infiltration and tenascin-C production were evaluated by HE and immunohistochemical staining. In the S/C group, considerable cells were observed in the core part of the graft, whereas in the S group, only a few cells could be observed in the graft at 4 weeks postoperatively (Figures 5(a) and 5(b)). At 16 weeks after surgery, fibroblast-like cells became more regular and denser in the S/C than in the S group (Figures 5(c) and 5(d)). The tenascin-C expression in the S group was obviously lower than that in the S/C group at 4 and 16 weeks after surgery (Figures 6(a) and 6(b)).

### 3.4. Graft–Bone Healing Assessment

**Figure 7:**
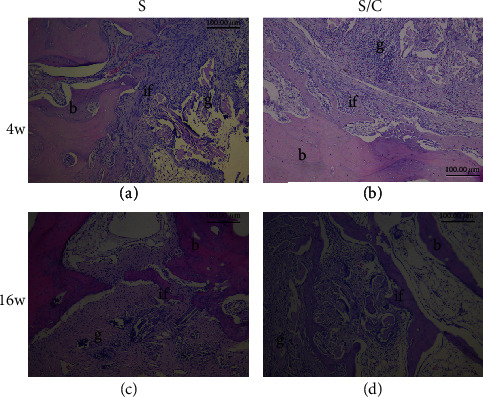
HE staining of the graft–bone interface for histological observation. At 4 weeks after the reconstruction surgery, no obvious bone integration was noticed in the two groups; although, more cells were distributed in the core part in the S/C group (b) than in the S group (a). At week 16, mature trabecular bone and considerable cell invasion in the scaffold could be noticed at the graft–bone interface in the S group (c). In the S/C group, integration of the trabecular bone into the graft was observed (d). g: graft; b: bone; if: interface.

**Figure 8:**
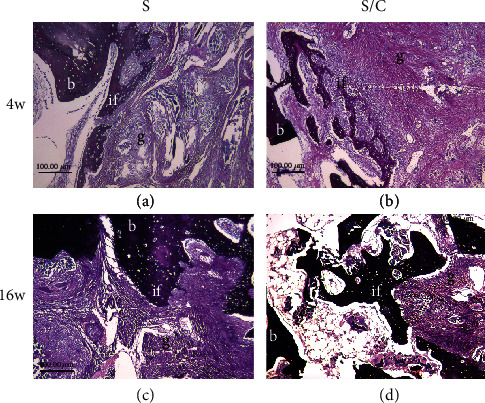
Russell–Movat staining of the graft–bone interface. At 4 weeks after the reconstruction surgery, no obvious bone integration was noticed in the two groups (a, b). At week 16, the mature trabecular bone could be noticed at the graft–bone interface in the S group (c), whereas in the S/C group, osteointegration with the trabecular bone into the graft was observed (d). g: graft; b: bone; if: interface.

Histological staining revealed connective tissue with a thin chondrocyte layer at the graft–bone interface at 4 weeks after the reconstruction surgery. No obvious bone integration was noticed in the two groups; although, more cells were distributed in the core part in the S/C group than in the S group (Figures 7(a) and 7(b); Figure 8(a) and 8(b)). By 16 weeks after surgery, the mature trabecular bone and considerable cell invasion in the scaffold could be noticed at the graft–bone interface in the S group, while integration with the trabecular bone in the graft was observed in the S/C group (Figures 7(c) and 7(d); Figures 8(c) and 8(d)).

### 3.5. Micro-CT Evaluations

**Figure 9:**
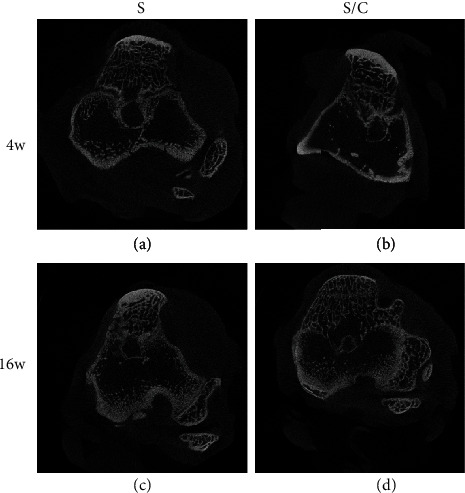
Representative micro-CT images. Few mineralized tissues were detected at the graft–bone interface at 4 weeks postoperatively (a, S group; b, S/C group). At 16 weeks postoperatively, distinct signals appeared indicating new mineralized tissue regeneration at the graft–bone interface of each group (c, S group; d, S/C group). The mineralized tissue signal and average bone tunnel area may indicate bone integration at the graft–bone interface.

Micro-CT reconstructed the high-resolution transverse sectional images of the tibia and femur. The formation of the mineralized tissue at the graft–bone interface could be easily observed. In both groups, few mineralized tissues were detected at the graft–bone interface at 4 weeks postoperatively (Figures 9(a) and 9(b)). BV/TV, Tb. Th, and BMD values were increased significantly more in the S/C than in the S group (Table 1). However, at 16 weeks after reconstruction surgery, distinct signals appeared indicating new mineralized tissue regeneration at the graft–bone interface in both groups (Figures 9(c) and 9(d)), with greater increases in Tb.Th and BMD observed in the S/C than in the S group (Table 1).

### 3.6. Biomechanical Test

**Figure 10:**
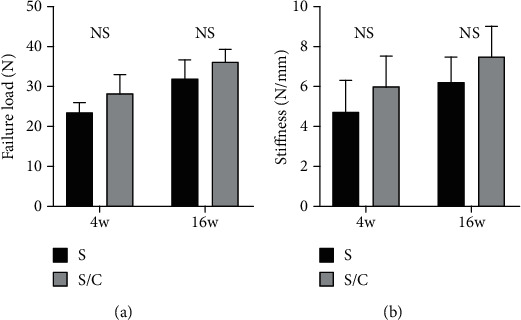
No significant differences were found in failure load (a) or stiffness (b) between the S group and S/C group at 4 and 16 weeks after the reconstruction surgery. NS indicates no significant difference between groups.

All grafts in both groups failed through rupture in the knee cavity or pullout from the bone tunnel. No obvious differences in the failure load were found between the two groups at 4 and 16 weeks after surgery (4 w, S 23.24 ± 2.18 vs. S/C 28.38 ± 4.07, *p* = 0.06; 16 w, S 31.85 ± 4.24 vs. 36.36 ± 2.58, *p* = 0.11; Figure 10(a)). Stiffness was calculated by recording the displacement and failure load from the load–deformation curve. The stiffness was not considerably different between the groups at 4 and 16 weeks after surgery (4 w, S 4.71 ± 1.42 vs. S/C 4.71 ± 1.42, *p* = 0.21; 16 w, S 6.18 ± 1.17 vs. 7.52 ± 1.31, *p* = 0.16; Figure 10(b)).

## 4. Discussion

Tissue engineering grafts for ACL reconstruction have focused on ligament regeneration in the knee cavity and bone integration at the graft–bone interface [31]. The present study revealed that BMSCs promoted ligament regeneration and graft–bone healing after reconstruction surgery using a silk–collagen scaffold. The scaffold was infiltrated by great many fibroblast-like cells and tenascin-C depositions at 4 and 16 weeks after surgery. The graft–bone interface exhibited good bone integration at 16 weeks after surgery. The results showed that BMSCs combined with silk–collagen scaffolds represent a good prospect in ACL tissue engineering and future clinical use.

Ideally, a tissue engineering scaffold for ACL reconstruction needs to simulate biological functions as well as the geometric structures of ligaments [32]. The ECM is important in guiding tissue ingrowth, maintaining homeostasis, and providing mechanical support during the ligament regeneration process. The silk–collagen scaffold takes advantage of silk's inherent mechanical properties and its suitability for knitting as well as the favorable biocompatibility of collagen matrix. Collagen matrix dominated the space between the silk fibroins and provided an attachment point for seeded cells.

The synovium layer covers the knee cavity, providing a less vascular microenvironment. At 4 and 16 weeks after surgery, fewer cells were attracted into the scaffold, and less ECM deposition occurred in the S group compared to the S/C group. According to the results, few cells migrated from surrounding tissues into the scaffold, and implanted BMSCs contributed proliferated cells to the scaffold and regenerated ECM. Fan and colleagues found that the production of tenascin-C, collagen-II, and collagen-I from stem cells was greatly improved after cocultivation with silk scaffolds after 7 and 14 days [33]. Tenascin-C, one of the extracellular matrix glycoproteins in intra-articular grafts, is always expressed in the actively remodeling tissue and has a highly restricted gene expression model [34]. The tenascin-C expression in the S group was obviously lower than that in the S/C group at 4 and 16 weeks postoperatively. Results demonstrated that grafts in the S/C group exhibited more vibrant ligament regeneration than did those in the S group, and implanted BMSCs contributed to cell proliferation and ECM deposition.

Successful ACL reconstruction requires solid graft–bone healing [17]. Graft–bone healing in the bone tunnel requires that bone grow inward into the graft–bone interface. Kanaya and colleagues [35] reported that transected sections shrank over time, and the interface in the MSC(-) group still lacked tissue at all points in time postoperatively, whereas in the MSC(+) group, GFP-positive cells were found at 2 and 4 weeks postoperatively in healing tissues covering the transected section. The histologic score of the MSC(+) group was obviously better than that of the MSC(-) group. As reported by Hong and colleagues, BMSCs may promote the graft–bone healing process, as cartilage-like cells proliferated and perpendicular collagen fibers increasingly formed at 4 weeks postoperatively in a rabbit model [36]. In the present study, cartilage-like cells proliferated, and less fibrocartilage-like tissue formed at the graft–bone interface in the S group than in the S/C group at 4 weeks after surgery. The mature trabecular bone was found in the core part of the graft in the S/C group at 16 weeks postoperatively, whereas only the mature trabecular bone was found at the interface in the S group. The graft–bone healing process may be promoted by the host stem cells from the circumambient bone marrow in the bone tunnel [37–39], but the host cell infiltration into the graft might take more than 4 weeks after surgery [20]. Based on the present study, it is considered that the implanted BMSCs mostly promoted bone integration between the graft and bone.

Oka and colleagues found that bone integration at the graft–bone interface determined micro-CT parameters [40]. Micro-CT could discern subtle changes in bone tunnels and collect gross information of newly formed mineralized tissue through imaging [41]. This study evaluated bone integration using micro-CT. More mineralized tissues were detected in the bone tunnel in the S/C group than in the S group at 4 and 16 weeks postoperatively; this finding corresponded to the histological findings.

The two main parameters of a ligament regenerated by tissue engineering are failure load and stiffness. Previous studies have shown that silk degraded via proteolytic degradation, resulting in a decrease in the scaffold mechanical strength [42, 43]. The speed of decrease in mechanical strength depends primarily on the physiological status, mechanical environment, implantation site, and scaffold structure. ECM including collagen fibers and proteoglycans could be produced by the infiltrated cells, which makes up for the mechanical strength decrease due to degradation. The mean failure load and stiffness in the S/C group were greater than those in the S group at 4 and 16 weeks after the procedure. The absence of a notable distinction between the two might be attributable to the small specimen dimensions.

One limitation of the present study was that we did not quantify the number of BMSCs implanted on the silk–collagen scaffold, and the optimal number of implanted cells remains unknown. Furthermore, the seeded BMSCs were not labeled and tracked, which represents another limitation. Although BMSCs seeded on the scaffold maintained good cellular morphology in vitro, the environments of the joint cavity and bone tunnel are different from that of a Petri dish. In a previous study, autologous BMSCs transfected with lentivirus vector expressing enhanced green fluorescent protein (Lv-eGFP) were seeded on a decellularized semitendinous tendon graft for ACL reconstruction [44]. The eGFP-positive cells could be observed at 12 weeks postoperatively; although, the eGFP-positive cell number at week 12 was significantly lower than that at week 4. The conclusions of the present study were based on cell infiltration and tenascin-C deposition and on graft–bone healing observed histologically. More data will be needed to confirm the fate of the implanted cells in a future study.

## 5. Conclusion

BMSCs may promote ligament regeneration in the cavity and bone integration at the graft–bone interface. Silk–collagen scaffold and BMSCs are very likely to be combined for clinical practice in the future.

## Figures and Tables

**Table 1:** Micro-CT evaluations (mean ± SD). BV/TV, Tb.Th, and BMD increased significantly more in the S/C group than in the S group at 4 weeks after reconstruction surgery. Significant increases in Tb.Th and BMD were observed in the S/C group relative to the S group. ^∗^ indicates a notable distinction between the two comparison groups.

Time point	Items	S	S/C	*p* value
4 w	BV/TV (%)	14.04 ± 0.41	15.53 ± 0.90	0.0169^∗^
	Tb.Th (mm)	0.27 ± 0.02	0.32 ± 0.02	0.0198^∗^
	Tb.N (1/mm)	0.24 ± 0.07	0.30 ± 0.01	0.0883
	Tb.Sp (mm)	1.21 ± 0.01	1.13 ± 0.07	0.0426^∗^
	BMD (mg/cm^3^)	0.08 ± 0.01	0.10 ± 0.00	0.0001^∗^

16 w	BV/TV (%)	22.29 ± 1.01	23.50 ± 0.98	0.1233
	Tb.Th (mm)	0.29 ± 0.05	0.36 ± 0.03	0.0406^∗^
	Tb.N (1/mm)	0.39 ± 0.10	0.47 ± 0.07	0.2725
	Tb.Sp (mm)	0.91 ± 0.03	0.83 ± 0.07	0.0887
	BMD (mg/cm^3^)	0.19 ± 0.02	0.22 ± 0.02	0.0494^∗^

## Data Availability

The data used to support the findings of this study are included within the article.
